# Off-label use information in electronic drug information resources

**DOI:** 10.5195/jmla.2022.1419

**Published:** 2022-10-01

**Authors:** Amanda Rothgeb, Robert D Beckett, Nadine Daoud

**Affiliations:** 1 AJRothgeb2022@manchester.edu, Staff Pharmacist, Kroger, Fort Wayne, IN. At the time the work was completed, Dr. Rothgeb was a student at Manchester University, Fort Wayne, IN.; 2 rdbeckett@manchester.edu, Clinical Standardization Coordinator, Parkview Health, Fort Wayne, IN. At the time the work was completed, Dr. Beckett was faculty at Manchester University, Fort Wayne, IN.; 3 nadz14@ymail.com, Staff Pharmacist, CAZMA, Dearborn, MI. At the time the work was completed, Dr. Daoud was a student at Manchester University, Fort Wayne, IN.

**Keywords:** Drug information, electronic databases, off-label uses

## Abstract

**Objective:**

To compare electronic drug information resources for scope, completeness, and consistency of off-label uses information, and to group resources into tiers based on these endpoints.

**Methods:**

An evaluation study of six electronic drug information resources (Clinical Pharmacology, Lexi-Drugs, American Hospital Formulary Service Drug Information, Facts and Comparisons Off-Label, Micromedex Quick Answers, and Micromedex In-Depth Answers) was conducted. All off-label uses for the top 50 prescribed medications, by volume, were extracted from all resources and used to determine scope (i.e., whether the resource listed the use). Fifty randomly selected uses were then evaluated for completeness (i.e., whether the entry cited clinical practice guidelines, cited clinical studies, provided a dose, described statistical significance, and described clinical significance) and consistency (i.e., whether the resource provided the same dose as the majority).

**Results:**

A sample of 584 uses was generated. The largest number of listed uses was in Micromedex In-Depth Answers (67%), followed by Micromedex Quick Answers (43%), Clinical Pharmacology (34%), and Lexi-Drugs (32%). The highest scoring resources for completeness were Facts and Comparisons Off-Label (median score 4/5), Micromedex In-Depth Answers (median score 3.5/5), and Lexi-Drugs (median score 3/5). Consistency with the majority in terms of dosing was highest for Lexi-Drugs (82%), Clinical Pharmacology (62%), Micromedex In-Depth Answers (58%), and Facts and Comparisons Off-Label (50%).

**Conclusion:**

The top-tiered resources for scope were Micromedex In-Depth and Quick Answers. For completeness, the top-tiered resources were Facts and Comparisons Off-Label and Micromedex In-Depth Answers. Lexi-Drugs and Clinical Pharmacology were the most consistent in dosing.

## INTRODUCTION

Off-label medication use is defined as prescription for a use which has not been approved by the U.S. Food and Drug Administration (FDA); administering the medication in an unapproved manner (e.g., when a medication is approved in a capsule formulation, but given as a solution); or administering the medication at an unapproved dose [1]. It is important to note that the FDA does not evaluate whether a medication is safe and efficacious for prescribed off-label uses. However, this does not necessarily mean that the drug is unsafe or inefficacious, and rigorous evidence from outside the new drug approval (NDA) process may exist. Manufacturers may not seek out FDA approval for all possible indications due to the cost of additional clinical trials for the new indication [2]. This cost may not be offset by the added income from relabeling the medication with the new FDA-approved indication.

Despite the lack of formal FDA approval, providers can prescribe drugs for an off-label use if they deem it appropriate for the patient [2]. Common reasons that drugs may be prescribed for unapproved uses include lack of approved medications for a condition and therapeutic failure of approved medications that have already been attempted. Off-label prescriptions account for roughly 10 to 20% of all prescriptions and are more commonly seen in very young and geriatric populations [3].

FDA prescribing information provides extensive information regarding all FDA-approved indications, but does not list potential off-label uses, or provide any information regarding these uses. Thus, other tertiary drug information resources are critical tools in the identification of potential off-label uses, in the evaluation of the usefulness and safety of a medication for off-label use, and for determining how a medication should be dosed and administered when used off-label. Considering the lack of published studies evaluating resources for off-label use content, this study sought to compare commonly used resources in terms of scope (i.e., whether the resource listed the use), completeness, and consistency, and to group resources into tiers based on these three endpoints.

## METHODS

This was a cross-sectional evaluation study of six electronic drug information resources that are commonly utilized by healthcare professionals to assist in off-label uses of medications. The drug information resources included in this study were: Clinical Pharmacology, Lexi-Drugs, American Hospital Formulary Service Drug Information (AHFS DI, available on Lexicomp Online), Facts and Comparisons Off-Label (available on Lexicomp Online), Micromedex Quick Answers, and Micromedex In-Depth Answers [4-9]. Because Micromedex Quick Answers directly links to content housed in In-Depth Answers, it was assessed for scope but not comprehensiveness. Anecdotally, the authors observed that Quick Answers and In-Depth Answers do not always index the same off-label uses.

To compare the off-label use content from these resources, a record was obtained of the Top 50 drugs, by prescription volume, as of October 1, 2020 [10]. This information was sourced from the Medical Expenditure Panel Survey (MEPS) prescribed medicines files released by the U.S. federal government. The record for each of the Top 50 drugs in each of the six drug information resources was reviewed by a single author to identify and extract each of the listed off-label uses; thus, a complete list containing each of the off-label uses for each drug across all six resources was compiled. Uses that were evaluated and graded by the resource (not the study investigators) as having insufficient evidence to justify the use or as being ineffective or unsafe were excluded from the study since some of the resources do not catalogue such uses. To measure the scope of each resource (i.e., the degree to which the resource covers the potential uses), the number and percentage of the remaining off-label uses addressed in each resource was determined. If resources used slightly different wording or definitions to describe similar uses, a second investigator was consulted to determine whether the uses were the same.

Once all potential off-label uses were identified, a random number generator was utilized to select 50 off-label uses, paired with the medication, for further analysis of completeness and consistency. Only uses that were described by at least three resources were placed in the random number generator. Micromedex Quick Answers was excluded from the analysis of completeness, as it simply provides a list of potential off-label uses with links to Micromedex In-Depth Answers for actual content. In order to evaluate completeness (i.e., whether the resource provides sufficient information in presenting the use), two investigators independently reviewed the randomly selected 50 uses to determine whether the entries 1) reference clinical practice guidelines, 2) reference clinical studies, 3) provide a use-specific dose, 4) describe the statistical significance of clinical studies, and 5) describe effect size measured in clinical studies. The number of citations (overall and specifically for clinical studies) was also gathered. Further evaluation of citation quality was beyond the scope of the study objective. Completeness elements were, on occasion, provided in other sub-databases in the resources but linked to the off-label use discussion. In those cases, linked information was included in the assessment. The two investigators jointly reviewed their collected data and resolved any differences by consensus. The number and percentage of entries containing each of the five completeness elements was determined for each resource. A completeness score (maximum of 5 points) was also calculated by summing results for each completeness element (1 point awarded for each). For example, if one resource cited clinical practice guidelines and clinical studies in support of the use, but did not address dosing, statistical significance, or effect size, it would receive 2 of 5 possible points for that use. If a resource did not contain an entry for a use that was randomly selected for evaluation, it was treated as a null rather than a score of 0 (i.e., it did not factor into the completeness score).

Consistency (i.e., the degree to which the resources are similar to each other) was measured using two endpoints, recommended dose and scope, as surrogate markers. These were selected for this purpose as they were straightforward to gather and simple to objectively compare among resources. Following collection of each dose for each use in each resource, the most common dose for each use among the resources was identified. If the dose in each resource aligned with the most common dose, it received a score of 1; if it did not, it received a score of 0. If no dose was present for the given use, then it was not included in this assessment. Similarly for scope, if the finding for the resource (i.e., entry or no entry) aligned with the majority of resources, it was considered to be consistent. Uses that were present in three resources were excluded from this calculation since a majority result could not be determined.

Data were analyzed in Microsoft® Excel and IBM® SPSS Statistics 24 [11,12]. Median and interquartile range (IQR) were used to describe continuous (given lack of normal distribution) and ordinal data. Number and percentage were used to describe categorical variables. Scope, completeness, and consistency were assessed using a hierarchical testing procedure to establish scoring tiers.

For each endpoint, the highest-scoring resource was placed in “Tier 1” and, in turn, compared to the next highest-scoring resource(s). If the difference between resources was found to be statistically significant (using an alpha value of 0.05), the lower-scoring resource formed a new tier (e.g., Tier 2). This resource was then compared, in turn, to subsequent lower-scoring resources until further new tiers were formed. This resulted in two to five tiers of resources formed for each endpoint. The Wilcoxon Signed-Rank and McNemar tests were used for ordinal and nominal data, respectively. Paired statistical tests were selected since the same off-label uses were evaluated in each resource.

## RESULTS

After reviewing all off-label uses listed for the 50 medications across the 6 resources, a total of 720 uses were identified. Of these, 136 were excluded from the study because they were identified as potential off-label uses where evidence was either inconclusive or ineffective, leaving a final sample of 584 uses. Most uses were represented in 1 (n=245, 42%), 2 (n=200, 34%), or 3 (n=66, 11%) resources. The most common categories of uses were cardiovascular (n=251, 43%), central nervous system (n=184, 32%), and endocrine (n=67, 11%). All remaining categories represented less than 5% of the sample.

The largest number of listed uses was in Micromedex In-Depth Answers (n=394, 67%), followed by Micromedex Quick Answers (n=252, 43%), Clinical Pharmacology (n=196, 34%), Lexi-Drugs (n=186, 32%), AHFS DI (n=100, 17%), and Facts and Comparisons Off-Label (n=35, 6%). When grouped into tiers by scope, Tier 1 consisted of Micromedex In-Depth Answers (p<0.001 vs. Tier 2), Tier 2 consisted of Micromedex Quick Answers (p<0.005 vs. Tier 3), Tier 3 consisted of Clinical Pharmacology and Lexi-Drugs (p<0.001 vs. Tier 4), Tier 4 consisted of AHFS DI (p<0.001 vs. Tier 5), and Tier 5 consisted of Facts and Comparisons Off-Label.

The 50 uses randomly selected for completeness and consistency evaluation are listed in Table 1. The most common categories of uses were central nervous system (n=18, 36%), cardiovascular (n=16, 32%), and endocrine (n=7, 14%). Completeness results are provided in Table 2. When allotted 1 point for each completeness element for a maximum score of 5, the highest scoring resource was Facts and Comparisons Off-Label (median score 4, IQR 4 to 5), followed by Micromedex In-Depth Answers (median score 3.5, IQR 2 to 4), Lexi-Drugs (median score 3, IQR 2 to 3), Clinical Pharmacology (median score 2, IQR 2 to 3), and AHFS DI (median score 2, IQR 1 to 3). When grouped into tiers, Tier 1 consisted of Facts and Comparisons Off-Label (p<0.05 vs. Tier 2), Tier 2 consisted of Micromedex In-Depth Answers (p<0.005 vs. Tier 3), Tier 3 consisted of Lexi-Drugs and Clinical Pharmacology (p<0.01 vs. Tier 4), and Tier 4 consisted of AHFS DI.

**Table 1:** Uses Analyzed for Completeness and aConsistency

Drug	Use	Resource				
		Clinical Pharmacology	Lexi-Drugs	Facts and Comparisons Off-Label	AHFS DI	Micromedex In-Depth Answers
Albuterol	Hyperkalemia	X	X	X		X
Amoxicillin	Anthrax	X	X		X	X
Amoxicillin	Endocarditis prophylaxis	X	X			X
Amoxicillin	Lyme disease	X	X			X
Amoxicillin	Periodontitis	X	X			X
Aspirin	Colorectal cancer prophylaxis	X	X		X	X
Aspirin	Kawasaki disease	X			X	X
Aspirin	Percutaneous coronary intervention	X	X		X	
Atenolol	Atrial fibrillation	X	X		X	
Atenolol	Paroxysmal supraventricular tachycardia prophylaxis	X	X		X	
Atorvastatin	Coronary artery disease prevention after transplant	X	X			X
Bupropion	Attention deficit-hyperactivity disorder	X	X	X	X	X
Carvedilol	Atrial fibrillation	X	X			X
Citalopram	Binge eating disorder		X	X		X
Citalopram	Obsessive compulsive disorder	X	X		X	X
Citalopram	Panic disorder	X	X		X	X
Citalopram	Premenstrual dysphoric disorder	X	X		X	X
Citalopram	Premature ejaculation		X	X	X	
Citalopram	Post-traumatic stress disorder	X	X	X	X	
Citalopram	Social anxiety disorder	X	X		X	
Duloxetine	Urinary incontinence	X	X	X	X	X
Fluoxetine	Premature ejaculation	X	X	X	X	X
Furosemide	Ascites	X	X			X
Furosemide	Hypertensive emergency	X				X
Gabapentin	Diabetic neuropathy	X	X	X	X	X
Ibuprofen	Gouty arthritis	X	X		X	X
Ibuprofen	Pericarditis		X	X	X	
Levothyroxine	Organ preservation	X	X			X
Lisinopril	Diabetic nephropathy	X			X	X
Lisinopril	Proteinuria	X	X			X
Metformin	Drug induced obesity	X	X	X		X
Metformin	Prediabetes	X	X			X
Metoprolol	Atrial fibrillation	X	X		X	X
Metoprolol	Migraine prophylaxis	X	X		X	X
Metoprolol	Supraventricular tachycardia		X		X	X
Omeprazole	Non-steroidal anti-inflammatory drug-induced ulcer prophylaxis	X	X			X
Pantoprazole	Duodenal ulcer	X			X	X
Pantoprazole	Gastroesophageal reflux disease	X			X	X
Pantoprazole	*H. pylori* eradication	X	X			X
Prednisone	Autoimmune hepatitis	X	X			X
Prednisone	Duchenne muscular dystrophy	X	X			X
Prednisone	Multiple myeloma	X	X			X
Prednisone	Pneumocystis pneumonia	X	X			X
Propranolol	Supraventricular arrhythmias	X			X	X
Propranolol	Thyrotoxicosis	X	X		X	X
Trazodone	Agitation associated with dementia		X	X		X
Trazodone	Insomnia	X	X	X		X
Venlafaxine	Hot flashes	X	X	X	X	X
Venlafaxine	Obsessive-compulsive disorder	X	X			X
Venlafaxine	Premenstrual dysphoric disorder	X	X	X		X
Total uses, n (%)		45 (90%)	44 (88%)	14 (28%)	27 (54%)	43 (86%)

* Presence of an entry indicated with an X

**Table 2:** Completeness Results

	Clinical Pharmaco logy (n=45)	Lexi - Dru gs (n=4 4)	Facts and Compari sons Off-Label (n=14)	AH FS DI (n=2 7)	Microm edex In-Depth Answers (n=43)
Describe s a clinical practice guideline, n (%)	24 (53%)	41 (93 %)	13 (93%)	12 (44 %)	19 (44%)
Describe s a clinical study, n (%)	26 (58%)	30 (68 %)	14 (100%)	16 (59 %)	32 (74%)
Provides a dose, n (%)	41 (91%)	44 (100 %)	9 (64%)	16 (59 %)	36 (84%)
Addresse s whether outcome s were statistical ly significa nt, n (%)	2 (4%)	0 (0%)	12 (86%)	0 (0%)	16 (37%)
Provides a specific effect size, n (%)	7 (16%)	0 (0%)	12 (86%)	4 (15 %)	26 (60%)
Overall complete ness score, median (IQR)	2 (2 to 3)	3 (2 to 3)	4 (4 to 5)	2 (1 to 3)	3.5 (2 to 4)
Citations, median (IQR)	2 (1 to 4)	4 (3 to 5.25)	6 (4.25 to 7.75)	3.5 (2 to 6.75)	3 (1 to 4.25)
Primary literature citations, median (IQR)	1 (0 to 2)	1.5 (0 to 2)	2 (2 to 2.75)	1 (0 to 4)	1 (0 to 2.25)

Following extraction and coding of dose information (when available), dose consistency was highest for Lexi-Drugs (36/44, 82%), followed by Clinical Pharmacology (28/45, 62%), Micromedex In-Depth Answers (25/43, 58%), Facts and Comparisons Off-Label (7/14, 50%), and AHFS DI (10/27, 37%). When grouped into tiers, Tier 1 consisted of Lexi-Drugs, Clinical Pharmacology, and Micromedex In-Depth Answers (p<0.01 vs. Tier 2), and Tier 2 consisted of Facts and Comparisons Off-Label and AHFS DI. Scope consistency was highest for Facts and Comparisons Off-Label (461/518, 89%), followed by AHFS DI (439/518, 85%), Lexi-Drugs (428/518, 83%), Clinical Pharmacology (415/518, 80%), Micromedex Quick Answers (361/518, 70%), and Micromedex In-Depth Answers (248/518, 48%) (518 was used as the denominator since a majority result could not be determined for the 66 uses that were present in 3 of the 6 resources). When grouped into tiers, Tier 1 consisted of Facts and Comparisons Off-Label (p<0.001 vs. Tier 2), Tier 2 consisted of AHFS DI, Lexi-Drugs, and Clinical Pharmacology (p<0.001 vs. Tier 3), Tier 3 consisted of Micromedex Quick Answers (p<0.001 vs. Tier 4), and Tier 4 consisted of Micromedex In-Depth Answers.

## DISCUSSION

Overall, the study identified that the strongest resources for off-label use content varied by endpoint. The top resources for scope were Micromedex In-Depth and Quick Answers. For completeness, the highest scoring resources were Facts and Comparisons Off-Label and Micromedex In-Depth Answers. Lexi-Drugs, Clinical Pharmacology, and Micromedex In-Depth Answers were the most consistent resources, specifically considering dosing recommendations; the non-Micromedex resources were very consistent in terms of scope. These results are generally similar to previous studies evaluating drug information databases for other types of information [13-15].

When evaluating scope results, it was interesting to note that most off-label uses considered in the study were only described by one or two resources. The scope scores observed in this study (6% to 67%) were lower than similar studies focused on drug information questions as a whole (53% to 81%), drug-drug interactions (67% to 97%), and dietary supplements (70% to 100%) [13,16,17]. However, this was somewhat similar to studies focused on drug-non-drug interactions [14,15]. For librarians and clinicians investigating off-label uses, this suggests that it is essential to check multiple resources in order to verify whether a use is supported for a particular drug. Though Facts and Comparisons Off-Label did not address a large portion of the sample, it was the most complete resource when it did address a particular use. It should be noted that inclusion of uses deemed insufficient to evaluate or ineffective would have led to even greater inconsistency of coverage.

Completeness scores also varied greatly by resource, with median scores ranging from 2 to 4 (out of 5 possible points), though it should be noted that these results are highly dependent on the specific elements selected for assessment of completeness (e.g., whether a guideline was described, whether a clinical study was described). Even within resources, there was high variability in terms of whether completeness elements were covered in the resource, with a couple exceptions related to statistical significance and effect size. These were both drivers of lower scores for several resources and could represent opportunities for improvement in off-label use content. Resources were generally well-cited, with the possible exception of Clinical Pharmacology.

Consistency scores ranged from 37% to 82% for dosing, which is widely variable but generally appears comparable to previous studies evaluating resources for drug-drug interactions (35% to 70%) and drug-non-drug interactions (15% to 87%) [13-15]. Dose was generally found to be a suitable surrogate for evaluating consistency, as it is typically objectively clear whether a resource is providing consistent dosing compared to others; however, there were several times when a second investigator was consulted because there were ambiguous results. These were usually related to dose ranges. This study sample was likely not large enough to detect meaningful differences in dose consistency among resources, given that resources did not always provide a use-specific dose. This sometimes prevented a “most common dose” from being defined, such as when only two resources provided a dose and those doses differed. For example, Lexi-Drugs (82%) and Clinical Pharmacology (62%) placed in the same tier despite widely different raw results. For scope, consistency was generally high in that whether a resource covered a specific use tended to align with the majority (mostly 80 to 89%), except for Micromedex In-Depth Answers. However, it should be noted that this database was less consistent because it was more likely to address uses not indexed in other databases.

Few previous studies have specifically evaluated the quality of information regarding off-label uses available in drug information resources, highlighting the importance of this study. One previous study investigated this topic by specifically examining antipsychotic off-label uses [18]. Considering similar endpoints to our study, the investigators found that Micromedex DrugDex (now In-Depth Answers), AHFS DI, and Clinical Pharmacology inconsistent results among resources.

There are some limitations to this study that should be noted. First, the lack of dosing completeness was not accounted for when projecting the necessary sample size for the study. However, the scope measurements consisted of a robust amount of data and the completeness score was well powered. One investigator was responsible for collecting data regarding the scope measurements, but this was very objective to gather. The number of resources included in this study was small, but all resources used in this study were considered the preferred Drug Information Databases and these resources were also present in *Basic Resources for Pharmacy Education*, July 2020 edition [19]. Finally, use of the Top 50 prescribed drugs to develop the sample resulted in over-representation of oral medications intended for chronic outpatient use, predominantly in cardiovascular, central nervous system, and endocrinology-related uses. Results may apply less to medications given by other routes in the inpatient setting; this may be an area for future study. Another future area of study could be examining pediatric- or geriatric-specific off-label uses described in specialty sub-databases, which was not a focus of this evaluation.

These findings are of value to librarians and clinicians, as they can help streamline searches based on needs. For example, if in-depth evaluation of efficacy of a medication for an off-label use is needed, Facts and Comparisons Off-Label would likely be the priority resource to examine. If a point-of-care user needs to quickly verify a prescribed dose, Lexi-Drugs or Clinical Pharmacology may be sufficient, though it should be noted that Micromedex sub-databases had higher scope scores and checking two sources may be necessary to locate information on a specific use. These results could also aid educators by providing evidence-based teaching pearls to help steer students toward the best resources based on the specific need. These results could help librarians justify budget expenditures, considering the low scope and consistency scores.

Overall, this study provides insight into the off-label use content of key electronic drug information databases. Results suggest that Micromedex and Lexicomp products (including the Facts and Comparisons Off-Label sub-database) generally provide the best content in this area, though sub-databases differ in their individual strengths and limitations.

## Data Availability

Data from this study are available upon request to the corresponding author.

## SUPPLEMENTAL FILES

**NA**
